# Screening Wild Birds for Tick-Borne Zoonotic Pathogens in Portugal

**DOI:** 10.3390/pathogens14010075

**Published:** 2025-01-15

**Authors:** Filipa Loureiro, João R. Mesquita, Luís Cardoso, Sérgio Santos-Silva, Guilherme Moreira, Jaqueline T. Bento, Vanessa Soeiro, Andreia Gonçalves, Filipe Silva, Patrícia F. Barradas, Ana C. Matos, Manuela Matos, Ana Cláudia Coelho

**Affiliations:** 1 Wildlife Rehabilitation Centre (CRAS), Veterinary Teaching Hospital (HVUTAD), University of Trás-os-Montes e Alto Douro (UTAD), 5000-801 Vila Real, Portugal; fsilva@utad.pt; 2 Animal and Veterinary Research Centre (CECAV), Associate Laboratory for Animal and Veterinary Sciences (AL4AnimalS), University of Trás-os-Montes e Alto Douro (UTAD), 5000-801 Vila Real, Portugal; lcardoso@utad.pt (L.C.); accoelho@utad.pt (A.C.C.); 3 School of Medicine and Biomedical Sciences (ICBAS), Porto University, 4050-313 Porto, Portugal; jrmesquita@icbas.up.pt (J.R.M.); up202110051@edu.icbas.up.pt (S.S.-S.); gmoreiravet@gmail.com (G.M.); jtbento@icbas.up.pt (J.T.B.); 4 Epidemiology Research Unit (EPIUnit), Instituto de Saúde Pública da Universidade do Porto, 4050-600 Porto, Portugal; patricia.barradas@iucs.cespu.pt; 5 Laboratory for Integrative and Translational Research in Population Health (ITR), 4050-600 Porto, Portugal; 6 Department of Veterinary Sciences, School of Agrarian and Veterinary Sciences (ECAV), University of Trás-os-Montes e Alto Douro (UTAD), 5000-801 Vila Real, Portugal; 7 Biological Park of Gaia (PBG), Rua da Cunha, 4430-812 Vila Nova de Gaia, Portugal; vanessasoeiro@cm-gaia.pt; 8 Wildlife Rehabilitation Centre of Santo André (CRASSA), Quercus ANCN, Moinho Novo, Galiza, 7500-022 Vila Nova de Santo André, Portugal; andreiagoncalves@quercus.pt; 9 TOXRUN—Toxicology Research Unit, University Institute of Health Sciences, CESPU, CRL, 4585-116 Gandra, Portugal; 10 Research Centre for Natural Resources, Environment and Society (CERNAS), Polytechnic Institute of Castelo Branco, 6000-767 Castelo Branco, Portugal; acmatos@ipcb.pt; 11 Quality of Life in the Rural World (Q-RURAL), Polytechnic Institute of Castelo Branco, 6001-909 Castelo Branco, Portugal; 12 Centre for the Research and Technology of Agro-Environmental and Biological Sciences (CITAB), University of Trás-os-Montes e Alto Douro (UTAD), 5000-801 Vila Real, Portugal; mmatos@utad.pt

**Keywords:** *Babesia*, *Borrelia*, *Hepatozoon*, PCR, piroplasms, Portugal, *Theileria*

## Abstract

Wild birds may be involved in the transmission of agents of infectious diseases, including zoonoses, a circumstance which raises a number of public and animal health issues. Migratory bird species play a significant role in the introduction of tick-borne pathogens to new geographic areas, contributing to the dissemination of various etiological agents. This preliminary study aimed to assess the occurrence of four potentially zoonotic pathogens (*Hepatozoon* spp., *Borrelia* spp., *Babesia* spp. and *Theileria* spp.) in the wild birds of Portugal. Blood and tissue samples were taken from 103 birds admitted at wildlife rehabilitation centers. Through the use of conventional PCR, our findings indicate no evidence of the circulation of these pathogens among the studied bird populations in the region. In the One Health context, it is relevant to understand how faraway avian populations play a role in the epidemiology of infectious diseases. Further molecular studies are needed to deepen the knowledge of avian piroplasmosis, borreliosis and hepatozoonosis.

## 1. Introduction

Birds are widely recognized as reservoirs of tick-borne pathogens, playing a key role in their transmission to new locations [1]. Some of these pathogens pose significant risks to human health, making it crucial to understand their distribution and epidemiology [2,3]. Several bacteria (*Anaplasma*, *Borrelia*, *Neoehrlichia*, *Rickettsia*), viruses (tick-borne encephalitis, Usutu, West Nile) and protozoan parasites (*Babesia*, *Hepatozoon*, *Theileria*, *Toxoplasma*) have already been isolated from ticks carried by birds or directly from avian individuals [2,4,5,6,7,8].

The genus *Hepatozoon* Miller, 1908 (Apicomplexa, Hepatozoidae) comprises cosmopolitan, arthropod-borne intracellular blood parasites frequently reported in many vertebrate hosts [9,10]. Although birds are not the most affected group, around 20 species of *Hepatozoon* have been identified in avian hosts [9,10]. Their life cycle is not yet fully understood, but infection most often occurs through the ingestion of infected blood-sucking invertebrates (ticks, mites, fleas and dipteran insects), in which gametogony and sporogony occur. Invertebrates will get infected with the blood meal from an infected vertebrate host. The parasites will infect erythrocytes and leucocytes [11,12].

*Borrelia burgdorferi* sensu lato is the etiological agent of Lyme disease (Lyme borreliosis) and is transmitted mainly by ticks of the genus *Ixodes* Latreille, 1795. These agents had already been isolated in arthropods infesting birds in the 1980s [13], and several reports have been registered since then [14,15,16]. Wildlife reservoirs are needed to maintain *B. burgdorferi* s.l. in nature, and birds (and mammals) vary in reservoir competence. Evidence of the involvement of a bird species as a carrier of this pathogen in Europe has been presented [17,18,19]. Competent reservoir hosts are capable of acquiring infection from vector ticks, allowing spirochetes to proliferate and be readily able to infect other vector ticks. There are reports from North America of a few bird species that were proven to be competent reservoirs of this bacterium, such as American Robins (*Turdus migratorius*), Northern Cardinals (*Cardinalis cardinalis*) and Song Sparrows (*Melospiza melodia*). The duration of infectivity is also a factor that influences transmission competence, with some species being highly infectious but only transiently so [20,21].

The genus *Babesia* Starcovici, 1893 (Apicomplexa, Babesiidae) comprises tick-borne parasites, which may be etiological agents of emergent zoonotic diseases [22]. Deep knowledge about their reservoirs and vectors is of paramount importance. The life cycle of these protozoa consists in asexual multiplication (merogony) in erythrocytes of the vertebrate host (including birds), and in sexual gametogony followed by asexual sporogony in the salivary glands of infected ticks (from both the Ixodidae and Argasidae families) [23]. *Theileria* is a closely related genus, and specimens of both genera are known as piroplasms. *Theileria* Bettencourt, França & Borges, 1907 (Apicomplexa, Theileridae) differs from *Babesia* in its ability to form schizonts, to colonize lymphocytes (besides erythrocytes) and to be transmitted only transstadially in ticks [24]. Furthermore, it has not yet been isolated in humans [23,25,26].

Portugal has a strategic geographical location and is a connection point chosen by many bird species for their migratory route between Europe and Africa. The country is the main migratory corridor in Western Europe, and millions of birds cross the Strait of Gibraltar twice a year to nest in the Iberian Peninsula and return to their wintering grounds [27,28]. Migration is influenced by many factors, including the evolution of food distribution, favorable climates and potential partners, but also by negative interactions such as with predators and pathogens [29,30]. In addition, the Portuguese region’s temperate climate offers refuge to several species of northern breeding birds [27].

Within the framework of a One Health approach, understanding the spillover of pathogens from wildlife to domestic animals and humans is critical. Enhanced epidemiological knowledge of these pathogens’ circulation provides valuable insights for managing and mitigating risks. This preliminary study aimed to assess the presence and species distribution of tick-borne pathogens capable of causing subclinical infection and clinical disease in wild birds.

## 2. Materials and Methods

### 2.1. Sample Collection

Blood samples (*n* = 74) were collected from wild birds that were admitted to three distinct wildlife recovery centers (WRCs) in mainland Portugal: the Wildlife Rehabilitation Centre of the Veterinary Teaching Hospital of University of Trás-os-Montes e Alto Douro (CRAS-HVUTAD), the Wildlife Rehabilitation Centre of Santo André (CRASSA) and the Biological Park of Gaia (PBG). All the birds were found lost, weakened or injured due to different causes, mostly anthropogenic ones (shotgun, electrocution, collision, etc.). Younger birds are also often found falling out of nests. Venipuncture was performed in the ulnar, jugular or metatarsal veins, according to the species’ anatomical features and the veterinarian’s preference. A volume between 0.3 and 0–5 mL was collected from each individual and immediately transferred to an EDTA tube. Samples were stored at −20 °C until further analysis.

Tissue samples (brain, cardiac muscle, kidney, liver, lung and spleen) (*n* = 29) were collected from dead birds during necropsy at CRAS-HVUTAD, stored in dry Eppendorf tubes and frozen at −20 °C until DNA extraction.

The databases of each center served as sources of information on the animals, including their age, geographical location and sex. A description of the samples tested is shown in Table 1.

Most of the birds (*n* = 52) sampled were below the adult age (nestling, fledgling or juvenile), 10 were classified as subadult and 30 as adult (mature). An age range was not registered for 11 individuals.

The geographical areas where the birds were found correspond to the following NUTS II regions: North (*n* = 48), Center (*n* = 2), Greater Lisbon (*n* = 1), Setúbal Peninsula (*n* = 2) and Alentejo (*n* = 43). Seven birds were classified as of unknown origin. The sex of 78 birds was not determined. Only 15 males and 10 females were identified. Of the represented species, most of the birds were resident in the Portuguese territory (*n* = 69), 14 were considered summer breeding visitors/residents, 13 were considered migratory summer breeding visitors, 5 were considered wintering visitors/residents, and 2 were considered exclusively wintering visitors.

### 2.2. DNA Extraction and Purification

DNA extraction from blood and organs was performed by adding a mixture of 420 µL of lysis buffer and 25 µL proteinase K solution and incubating at 37 °C for 10 min. Afterward, incubation samples were briefly vortexed and centrifuged for 2 min at 6000× *g*. After centrifugation, 140 μL of the supernatant was used for DNA extraction and purification with the QIAamp DNA Mini Kit (Qiagen, Hilden, Germany), following the manufacturer’s instructions. The extraction was automated using the QIAcube^®^ platform (Qiagen, Hilden, Germany). The purified DNA was then stored at −20 °C in RNase-free water until further analysis.

### 2.3. Molecular Detection of Zoonotic Pathogens

The detection of *Hepatozoon* spp. was performed with the set of primers Hep F/Hep R, targeting the 18S SSU rRNA element and amplifying a fragment of 800 bp [31]. The detection of *Borrelia* spp. was performed using the primer set M1/M2, targeting the highly conserved 357/358 bp segment of the borrelial 16S rRNA gene [32]. The detection of piroplasms was performed using oligonucleotide primers targeting the highly conserved 408 bp segment of the small subunit ribosomal DNA [33]. Positive controls for all targets were derived from previously identified isolates in our lab [34], including a *Borrelia* sample with accession number PQ682435.

### 2.4. General Procedures

All PCR procedures were run on a T100 thermocycler (Bio-Rad; Hercules, CA, USA). Each reaction mixture had a total volume of 20 μL, containing 0.4 μM of each primer, 10 μL of Speedy Supreme NZYTaq 2× Green Mastermix (NZYTech^®^, Lisboa, Portugal), 5.5 μL of RNase-free water and 5 μL of template DNA, prepared according to the manufacturer’s instructions. The amplified DNA fragments were subjected to electrophoresis at 100 V for 40 min on 1.5% agarose gels stained with Xpert Green Safe DNA gel dye (GriSP^®^, Porto, Portugal), and the results were confirmed under UV light.

## 3. Results and Discussion

A total of 103 samples (74 blood samples and 29 mixed organ samples) were tested by PCR for *Hepatozoon* spp., *Borrelia* spp., *Babesia* and *Theileria* spp. Results were negative for all four agents. Two of the samples exhibited a band of the expected size in the gel, suggesting the presence of *Borrelia* in the corresponding assay. However, sequencing revealed only the host’s DNA. All the results were negative for the other three genera.

*Hepatozoon* spp. infect all groups of terrestrial vertebrates, and their infections in domestic animals have frequently been reported across Europe [35,36,37,38]. *Hepatozoon* spp. infecting birds have also been reported, infecting either erythrocytes or leucocytes [9,11,39,40]. Both the Ixodidae and Argasidae tick families are involved in transmitting these agents, as well as fleas, mosquitoes and other arthropods. In vertebrate hosts, *Hepatozoon* merogony occurs in different tissues (e.g., liver, spleen, bone marrow, lymph nodes and intestine), but some species’ cycles are not yet fully elucidated, as is the case for *Hepatozoon* in avian species. Several species have been identified, but recent studies have found that some are more similar to amphibian parasites, namely, *Lankestrella* spp. [10,41,42]. Our results, which showed that all of our wild birds tested negative for *Hepatozoon* spp., contribute valuable information to this field. While *Hepatozoon* spp. infections have been documented across a range of vertebrates, including birds, the absence of detectable infections in our sample highlights the potential for regional or ecological variability in the prevalence of these parasites. This underscores the importance of further molecular and prevalence studies in wild avian populations to clarify the distribution of *Hepatozoon* spp. and their role in avian health.

Lyme disease is the most common vector-borne disease in the temperate regions of the Northern Hemisphere, caused by *B. burgdorferi* s.l. bacteria transmitted by *Ixodes* ticks. In North America, *B. burgdorferi* sensu stricto is the etiological agent, but worldwide, the *B. burgdorferi* s.l. spirochete complex is mainly responsible for causing disease. Subadult ticks (larvae or nymphs) ingest spirochetes while feeding upon a bacteremic host and may then infect other vertebrate hosts while feeding during subsequent life stages (nymphs or adults, respectively) [43,44]. Although the role of birds as alternative hosts and reservoirs of *B. burgdorferi* s.l. and their involvement in the transmission cycle have not yet been definitively established, it is widely acknowledged that birds likely contribute to the maintenance of *B. burgdorferi* s.l. in nature. Furthermore, they may facilitate long-distance dispersal by carrying infected ticks and are increasingly recognized as an underestimated component of Lyme disease ecology [13,18,45]. *B. burgdorferi* s.l. is the most prevalent species in southwestern Europe, with the genospecies *B. lusitaniae* (also present in North Africa) being the most commonly encountered in Portugal. This agent has already been isolated from ticks parasitizing different species of the order Passeriformes. In Western and Central Europe, *Turdus* (Linnaeus, 1758) has been identified as a genus that seems to play an important role in keeping *Borrelia* spp. in circulation [43,46,47]. In the present study, only four *Turdus* individuals were tested, and all were negative for *B. burgdorferi*, consistent with the negative results observed in all other samples.

Avian infecting piroplasms are largely under-studied compared to other hemoparasites, like *Haemoproteus* spp., *Leucocytozoon* spp. or *Plasmodium* spp. In contrast, of the majority of species that infect domestic mammals and are responsible for causing disease, only two avian species, *Babesia shortti* and *Babesia uriae*, are recognized as pathogenic. *Babesia* infection has already been reported in several orders of birds (Charadriiformes, Passeriformes, Procellariiformes and Sphenisciformes, among others) from different parts of the world [48,49,50,51]. Some studies have reported that the prevalence of *Babesia* infection in birds is relatively low [24,52], but others revealed a relevant proportion of animals positive for piroplasmid-specific 18S rDNA [53]. Juveniles seem to be more susceptible to *Babesia* infections compared to adults, with the latter likely exhibiting greater resistance due to factors such as advancing age and the development of acquired immunity. Additionally, immunosuppressed individuals are more vulnerable to infection, as their compromised immune systems are less able to mount an effective defense [52].

Hemoparasites are transmitted through various vectors (e.g., ticks, mosquitoes and fleas), and their transmission to birds is largely influenced by factors such as habitat type and population density. While seabirds are often considered to be relatively free from blood parasites, this perception may be influenced by specific ecological conditions or gaps in surveillance [52]. On the other hand, colonial seabirds may be particularly exposed to vectors during the reproduction season, because of their tendency to breed in high densities and reuse the same colony locations during consecutive years. Yellow-legged gulls (*Larus michahellis*) are one of the species in which *Babesia* spp. have been described [50], with notable representation in this study in Southern France, in which it yielded only negative results.

Regarding the variables analyzed, we relied on available information concerning the locations where the birds were found and rescued. It is important to note that there may be intermediate individuals for which age and/or sex cannot be reliably determined based solely on phenotypic characteristics. Many species still lack comprehensive data on this topic. Sex determination is even more complex than age estimation, as the methods used to sex birds vary across species, often influenced by age and season [54,55]. Furthermore, while the DNA extraction process ensured high-quality isolation, we acknowledge the limitation that viability testing to confirm the DNA’s origin from live pathogens was not performed, which may have influenced the interpretation of our results regarding pathogen presence. Considering the influence of age on the prevalence of blood parasites and other pathogens, an observed age dependency has been reported (juveniles are infected less, adults more), especially evident in long-distance migrating species. This could be related to the tolerance of migratory birds toward these parasites. Knowledge of the age of migratory birds and resident birds in specific geographical areas is a significant variable in determining not only the distribution but also the transmission of parasites in the regions studied [56]. The probability of infection increases as birds get older [57].

The term resident bird describes species that spend the whole year at their breeding grounds. Migratory birds fly south or west. It is widely demonstrated that birds can carry pathogens or infected vectors during migratory movements, which can be transmitted between species wherever there is a higher concentration of individuals [58,59]. Migration and infection can interact in several ways, and evaluating the migratory status of birds in relation to pathogen detection is essential for understanding their potential role in the epidemiology of various diseases. Migrating species face a higher infection risk than species that do not migrate, and the increased circulation of infected individuals can lead to the spread of diseases that would otherwise be localized [30]. Specifically, vector-borne diseases, migration or dispersion movements may lead to an increased risk of exposure to different tick species and potential contact with other hosts [60]. On the other hand, it has already been suggested that seasonal migration can drastically reduce the prevalence of pathogens in animal populations and that the presence of a pathogen can alter the migratory strategy that maximizes the size of the host population [61]. For other pathogens, like *Babesia* spp., many studies report infection in immature birds, suggesting that infection is probably acquired very early, in the nest. In adult birds, chronic infectious disease is more frequently related to depressed immune status [62]. An early prediction of how migratory patterns are likely to shift in the future is already being studied [29]. In this study, no pathogens were detected, which provides important information about the absence of these pathogens in the sampled population. Our study offers a robust geographical sample, though further research is needed to expand on these findings and explore additional factors influencing pathogen dynamics in avian species.

Exposure to ticks depends on the behavior of species at different levels. Ground-feeder birds spend a predominant part of their lifespan in potential tick habitats and have a higher chance of being infested with ticks than birds that have other feeding behaviors. In turn, sedentary birds are more susceptible to tick exposure than migratory birds, and short-distance migratory birds are more prone to being exposed to ticks than long-distance migratory birds [63,64]. In this study, unfortunately, no information was available on when the birds were sampled. This could be relevant, because climatic variables, especially temperature fluctuations, significantly affect the distribution, survival and foraging behavior of ticks [64]. It is important to highlight that the prevalence of ticks in birds admitted to the WRCs is generally low, including within the population studied here. Furthermore, the detection of infection in feeding ticks or their hosts does not necessarily indicate that these hosts serve as competent reservoirs for the pathogens. This distinction is critical for understanding the role of birds in the transmission and maintenance of tick-borne disease [18]. This study only included descriptive statistics, as a relatively small proportion of the animals had complete data for all variables under analysis, and all results were negative. As a result, it was not possible to assess potential predisposing factors. The tested birds originated from wildlife rehabilitation clinics and do not represent a random or unbiased sample of wild bird populations. As such, estimating prevalence or conducting a power analysis would not yield reliable results for broader wild bird populations. This highlights the need for further investigation, suggesting that additional variables should be considered in future studies to better understand the underlying factors involved.

## 4. Conclusions

We employed PCR to assess the prevalence of *Hepatozoon*, *Borrelia* and piroplasms in wild birds rescued in Portugal. All results were negative in this preliminary study, suggesting that, at present, wild birds do not seem to pose a significant threat to animal or public health regarding the transmission of these pathogens, but more comprehensive studies are needed to accurately assess the presence and distribution of these pathogens in wild birds in Portugal using a larger and more representative number of samples. Indeed, the sample in this study was relatively small, and the biological material (blood or tissues) used to detect the agents was not determined specifically for each of them in the design of the study. Further research is essential to deeply evaluate the role of avian species in the epidemiology of these pathogens.

## Figures and Tables

**Table 1:** List of individuals tested, organized by order, family, species and wildlife rehabilitation center (WRC).

Order	Family	Species	CRAS-HVUTAD *n* (%)	CRASSA *n* (%)	PBG *n* (%)
Accipitriformes	Accipitridae	*Accipiter gentilis* (Northern goshawk)	1 (1.0)	0 (0)	2 (1.9)
		*Accipiter nisus* (Sparrowhawk)	2 (1.9)	0 (0)	1 (1.0)
		*Buteo buteo* (Common buzzard)	4 (3.9)	1 (1.0)	0 (0)
		*Circus pygargus* (Montagu’s harrier)	2 (1.9)	0 (0)	0 (0)
		*Gyps fulvus* (Griffon vulture)	5 (4.9)	1 (1.0)	0 (0)
		*Hieraaetus pennatus* (Booted eagle)	2 (1.9)	0 (0)	0 (0)
		*Milvus migrans* (Black kite)	2 (1.9)	1 (1.0)	0 (0)
Anseriformes	Anatidae	*Anser anser* (Greylag goose)	0 (0)	1 (1.0)	0 (0)
Apodiformes	Apodidae	*Apus apus* (Common swift)	1 (1.0)	0 (0)	0 (0)
		*Apus pallidus* (Pallid swift)	2 (1.9)	0 (0)	0 (0)
Bucerotiformes	Upupidae	*Upupa epops* (Eurasian hoopoe)	0 (0)	1 (1.0)	0 (0)
Charadriiformes	Laridae	*Larus fuscus* (Lesser black-backed gull)	0 (0)	4 (3.9)	1 (1.0)
		*Larus michahellis* (Yellow-legged gull)	0 (0)	24 (23.3)	1 (1.0)
		*Egretta garzetta* (Little egret)	0 (0)	0 (0)	1 (1.0)
Ciconiiformes	Ciconiidae	*Ciconia ciconia* (White stork)	3 (2.9)	10 (9.7)	0 (0)
Columbiformes	Columbidae	*Columba livia* (Domestic pigeon)	3 (2.9)	0 (0)	1 (1.0)
		*Columba palumbus* (Common woodpigeon)	3 (2.9)	0 (0)	0 (0)
		*Streptopelia decaocto* (Eurasian collared dove)	2 (1.9)	0 (0)	0 (0)
Cocariiformes	Meropidae	*Merops apiaster* (European bee-eater)	2 (1.9)	0 (0)	0 (0)
Passeriformes	Corvidae	*Corvus corone* (Carrion crow)	0 (0)	1 (1.0)	0 (0)
		*Garrulus glandarius* (Eurasian jay)	2 (1.9)	0 (0)	0 (0)
	Hirundinidae	*Delichon urbicum* (Western house martin)	1 (1.0)	0 (0)	0 (0)
	Muscicapidae	*Erithacus rubecula* (European robin)	1 (1.0)	0 (0)	0 (0)
	Passeridae	*Passer domesticus* (House sparrow)	2 (1.9)	0 (0)	0 (0)
	Turdidae	*Turdus merula* (Blackbird)	3 (2.9)	0 (0)	0 (0)
		*Turdus philomelos* (Song thrush)	1 (1.0)	0 (0)	0 (0)
Strigiformes	Strigidae	*Athene noctua* (Little owl)	0 (0)	1 (1.0)	0 (0)
		*Bubo bubo* (Eurasian eagle owl)	3 (2.9)	0 (0)	0 (0)
		*Strix aluco* (Tawny owl)	2 (1.9)	0 (0)	0 (0)
	Tytonidae	*Tyto alba* (Barn owl)	1 (1.0)	0 (0)	0 (0)
			50 (48.5)	46 (44.7)	7 (6.8)
		Total	103 (100)		

*n*—Number of samples.

## Data Availability

The data presented in this study are available on request from the corresponding author.
